# Difficulty differentiating primary mediastinal classical Hodgkin lymphoma from inflammatory myofibroblastic tumor: A case report

**DOI:** 10.1111/1759-7714.15197

**Published:** 2023-12-30

**Authors:** Kentaro Akata, Kei Yamasaki, Yosuke Chiba, Takako Kawaguchi, Hiroki Dosaka, Toshiki Morimoto, Yasuyuki Higashi, Chinatsu Nishida, Shohei Shimajiri, Kazuhiro Yatera

**Affiliations:** ^1^ Division of Infection Control and Prevention University of Occupational and Environmental Health, Japan Kitakyushu Japan; ^2^ Department of Respiratory Medicine University of Occupational and Environmental Health, Japan Kitakyushu Japan; ^3^ Department of Environmental Health Engineering Institute of Industrial Ecological Sciences, University of Occupational and Environmental Health, Japan Kitakyushu Japan; ^4^ Department of Pathology University of Environmental and Occupational Health, Japan Kitakyushu Japan

**Keywords:** Hodgkin lymphoma, inflammatory myofibroblastic tumor, malignant lymphoma

## Abstract

A 20‐year‐old Japanese man visited our hospital because an enlarged mediastinal shadow had been detected on chest x‐ray. Chest computed tomography revealed a large mediastinal mass with multiple lymph node enlargement, pericardial effusion, and bilateral pleural effusion. He was diagnosed with inflammatory myofibroblastic tumor (IMT) based on a thoracoscopic tumor biopsy. Initial corticosteroid and celecoxib treatment was only partially effective; therefore, additional tumor rebiopsy and left axillary lymph node biopsy were performed. Based on the findings, the patient was rediagnosed with classical Hodgkin lymphoma (CHL). To date, there has only been one report of a case initially diagnosed as IMT and rediagnosed as CHL, as in our case, and only three reports of malignant lymphoma mimicking IMT. When IMT is suspected based on pathological findings and subsequently with treatment failure, possible CHL and performing rebiopsy should be considered.

## Graphical abstract

We present a rare case initially diagnosed as inflammatory myofibroblastic tumor (IMT) and rediagnosed as classical Hodgkin lymphoma (CHL) on second biopsy. In the pathological lesions of patients with CHL, Hodgkin (arrowhead) and Reed/Sternberg (HRS) (arrow) cells are surrounded by reactive cells such as lymphocytes, macrophages, eosinophils, mast cells, plasma cells, stromal cells, fibroblasts, and other cell types. Therefore, it may occasionally be difficult to differentiate CHL from IMT, when stromal cells and fibroblasts are abundantly present. In patients with IMT with unfavorable treatment response, it is important to consider possible CHL and perform an additional biopsy for proper diagnosis.

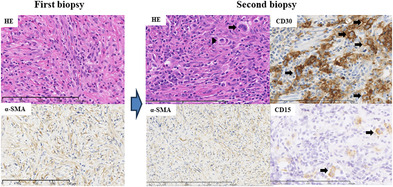

## INTRODUCTION

Classical Hodgkin lymphoma (CHL) is a rare lymphatic system malignancy.^1^ Malignant lymphoma (ML), including CHL, accounts for approximately 20% of mediastinal tumors.^2^, ^3^ Inflammatory myofibroblastic tumor (IMT) is characterized by myofibroblastic spindle cells along with inflammatory cell infiltration.^4^ Given that CHL induces pronounced inflammatory alterations in tumor and surrounding tissues,^5^ leading to fibrosis as seen in IMT,^6^, ^7^, ^8^, ^9^, ^10^, ^11^, ^12^ accurate CHL diagnosis may be difficult because of extensive fibrotic areas in CHL.^13^ To date, there has been only one case report of CHL mimicking IMT^14^ and only three reports of ML mimicking IMT.^15^, ^16^ Here, we present a rare case initially diagnosed as IMT and rediagnosed as CHL on second biopsy.

## CASE REPORT

**FIGURE 1:**
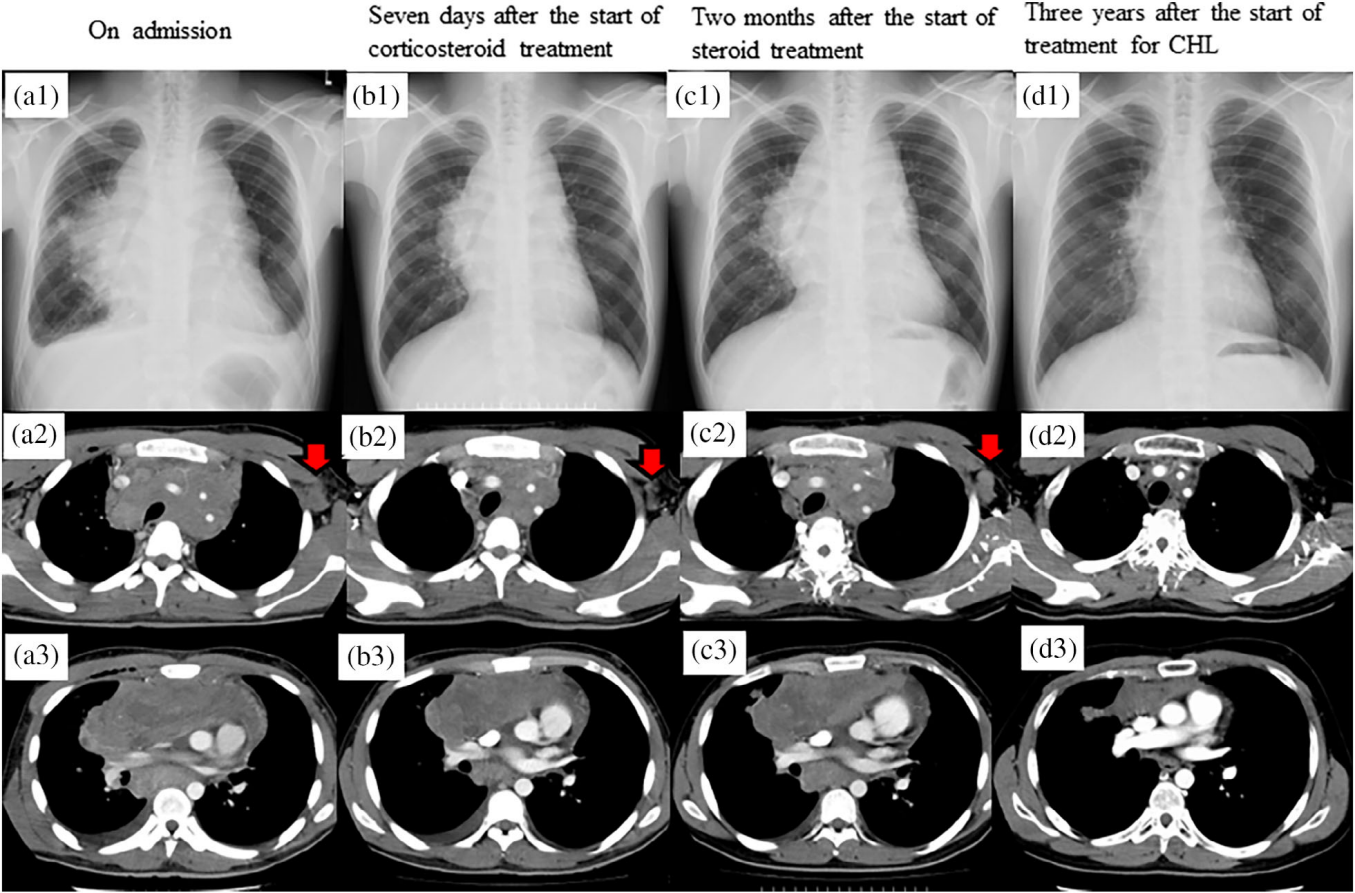
(a) Chest x‐ray film (a1) and computed tomography (CT) images (a2 and a3) on admission, showing a large mediastinal mass with pericardial and bilateral pleural effusions and left axillary lymphadenopathy (red arrow). (b) Chest x‐ray (b1) and CT images (b2 and b3) seven days after the start of corticosteroid treatment, showing shrinkage of the mediastinal tumor and lymph nodes (red arrow). (c) Chest x‐ray film (c1) and CT images (c2 and c3) two months after the start of corticosteroid treatment, showing regrowth of the mediastinal tumor and lymph nodes (red arrow). (d) Chest x‐ray film (d1) and CT images (d2 and d3) three years after the start of treatment for CHL, showing mediastinal tumor shrinkage and lymph node enlargement disappearance. CHL, classical Hodgkin lymphoma.

**FIGURE 2:**
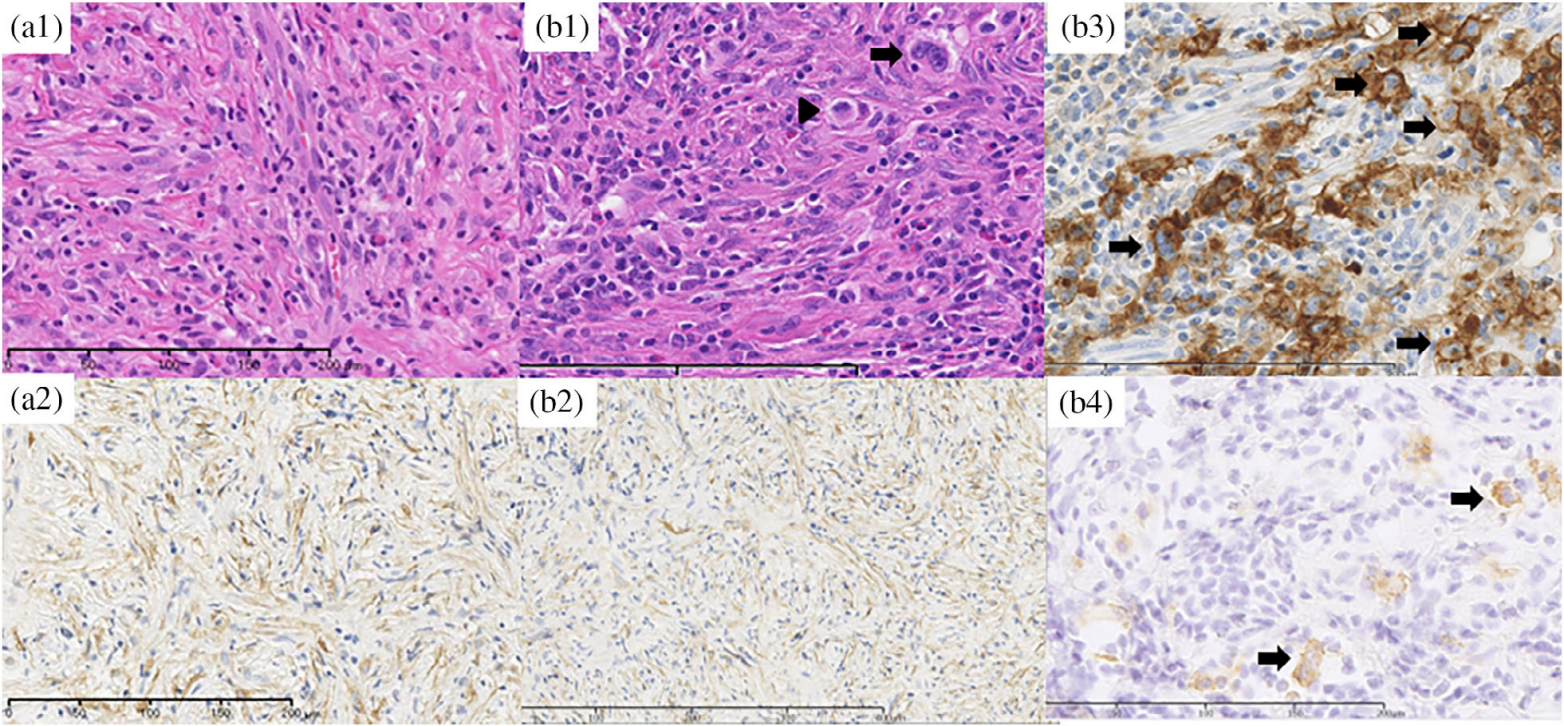
Histological findings on first biopsy (a1), showing that the tumor is composed of spindle cells arranged in a storiform pattern, with variable inflammatory cells mainly composed of lymphocytes (hematoxylin–eosin stain, high‐power field). Immunohistochemical staining of α‐SMA (a2), showing that the spindle cells were positively stained (high‐power field). Histological findings on second biopsy (b1), showing that the tumor is composed of HRS cells, including Hodgkin (arrow head) and Reed‐Sternberg (arrow) cells, surrounded by spindle cells in a storiform pattern with inflammatory cells mainly composed of lymphocytes (hematoxylin–eosin stain, high‐power field). Immunohistochemical staining of α‐SMA (b2), showing that the spindle cells were positively stained (high‐power field). Immunohistochemical staining of CD30 and CD15 (b3, b4), showing that HRS cells (arrow) were positively stained (high‐power field). α‐SMA, α‐smooth muscle actin; He, hematoxylin–eosin; HRS, Hodgkin and Reed/Sternberg.

A 20‐year‐old healthy Japanese man with a history of childhood asthma presented with a dry cough. One month later, he developed dyspnea and night sweats and was treated for asthma, but his symptoms did not improve. Two months later, an enlarged mediastinal shadow was detected on a chest x‐ray, and he was subsequently transferred to our hospital. The patient exhibited marked edema from the face to the neck and on both upper extremities, with jugular vein distention. Chest auscultation revealed coarse crackles in both lung fields. Laboratory findings on admission (Table 1) revealed elevated serum C‐reactive protein (CRP; 7.11 mg/dL) and soluble interleukin‐2 receptor (1563 U/mL) levels. A chest x‐ray film (Figure 1a1) revealed an enlarged mediastinal shadow, and chest computed tomography (CT) (Figure 1a2,a3) showed a 15 × 10‐cm mass from the anterior mediastinum to the middle mediastinum, with bilateral supraclavicular lymph node enlargement, #7 and #12R mediastinal lymph nodes, and left axillary lymph node, as well as pericardial and bilateral pleural effusions. Thoracoscopic anterior mediastinal tumor biopsy was performed under local anesthesia, and histopathological examination revealed proliferation of spindle‐shaped cells negative for anaplastic lymphoma kinase (ALK) but positive for α‐smooth muscle actin, and inflammatory cell infiltration (Figure 2a1,a2). Based on these pathological findings, he was diagnosed with IMT. Surgical removal of the tumor was difficult because it surrounded blood vessels. Therefore, corticosteroid and celecoxib therapy was started and was initially effective (Figure 1b), but the tumor continued to grow (Figure 1c). Secondary thoracoscopic mediastinal tumor biopsy and left axillary lymph node biopsy were performed, and histopathological examination revealed the presence of Hodgkin and Reed/Sternberg (HRS) cells positive for CD30 and CD15 amidst a background of spindle cells (Figure 2b1–b4), diagnostic for stage IV nodular sclerosing CHL (Ann Arbor classification). On the 112th day, the patient was transferred to the hematology department to receive systemic chemotherapy, and his tumor shrunk after three years of chemoradiotherapy (Figure 1d1–d4). He is currently alive, seven years after autologous stem cell transplantation completion.

**TABLE 1:** Laboratory findings on admission.

Hematology		Serology	
WBC	9,400/μL	hCG	1.2 mIU/mL
Neu	78.8%	AFP	0.7 ng/mL
Lym	4.5%	CEA	1.4 ng/dL
Mon	13.4%	CYFRA	0.5 ng/dL
Eos	0%	ProGRP	35 pg/dL
Bas	0.1%	IgG	789 mg/dL
RBC	573 × 10^4^/μL	sIL‐2R	1563 U/mL
Hb	16.0 g/dL	EB VCA‐IgM	<10
Hct	48.4%	EB VCA‐IgG	<10
Plt	28.2 × 10^4^/μL	EBNA	<10
Biochemistry		Blood gas analysis (O_2_ 3L/min, nasal)	
TP	5.6 g/dL	pH	7.42
Alb	2.7 g/dL	PaO_2_	111 Torr
T‐Bil	0.4 mg/dl	PaCO_2_	38 Torr
AST	13 IU/L	HCO_3_^−^	24.6 mmol/L
ALT	12 IU/L	BE	0.4 mmol/L
ALP	184 IU/L		
γ‐GTP	21 IU/L		
LDH	184 IU/L		
BUN	9 mg/dL		
Cr	0.67 mg/dL		
Na	136 mEq/L		
K	4.3 mEq/L		
Cl	101 mEq/L		
CRP	7.11 mg/dl		

Abbreviations: AFP, alpha fetoprotein; Alb, albumin; ALP, alkaline phosphatase; ALT, alanine aminotransferase; AST, aspartate aminotransferase; BE, base excessBUN, blood urea nitrogen; CEA, carcinoembryonic antigen; Cre, creatinine; CRP, C‐reactive protein; hCG, human chorionic gonadotropin; CYFRA, cytokeratin 19 fragment; EBNA, Epstein–Barr virus‐nuclear antigen antibody; EB VCA, Epstein–Barr virus viral‐capsid antigen; Hb, hemoglobin; HCO_3_^−^, hydrogencarbonate; Ht, hematocrit; IgG, immunoglobulin G; LDH, lactate dehydrogenase; Pro GRP, progastrin‐releasing peptide; PaO_2_, partial pressure of oxygen in arterial blood; PaCO_2_, partial pressure of carbon dioxide in arterial blood; pH, power of hydrogen; PLT, platelet; RBC, red blood cell; sIL‐2R, soluble interleukin 2 receptor; T‐Bil, total bilirubin; TP, total protein; γ‐GTP, gamma‐glutamyl transferase; WBC, white blood cell.

## DISCUSSION

Our patient represents a rare case of rediagnosis of CHL after initial IMT diagnosis. HL occasionally mimics IMT^6^, ^7^, ^8^, ^9^, ^10^, ^11^, ^12^ because of pronounced inflammatory alterations in tumor and surrounding tissues^5^ that leads to fibrosis as observed in our patient, and an accurate diagnosis of CHL may sometimes be difficult because of extensive fibrotic areas in the pathological lesions. When the pathological findings suggest IMT and IMT treatment is ineffective, it is important to consider the possibility of ML and perform additive biopsy to elucidate proper pathological diagnosis.

In the pathological lesions of patients with CHL, HRS cells are surrounded by reactive cells such as lymphocytes, macrophages, eosinophils, mast cells, plasma cells, stromal cells, fibroblasts, and other cell types. Therefore, it may occasionally be difficult to differentiate CHL from IMT, when stromal cells and fibroblasts, which are instigated by interleukin‐13, tumor necrosis factor‐alpha, transforming growth factor‐beta, and other mediators secreted by HRS cells,^5^ are abundantly present. To date, only three cases of ML mimicking IMT have been reported (Table 2).^14^, ^15^, ^16^ In Case 2, a buccal mucosa mass was excised, leading to the diagnosis of IMT.^16^ Cases 1 and 3 were initially diagnosed with IMT based on initial biopsy results, as in our case. Their tumors proliferated despite treatment for IMT, and second biopsies revealed ML. In Case 1, the tumor completely vanished after IMT treatment, suggesting that the tumor emerged after IMT treatment discontinuation.^15^ In contrast, our patient presented CHL mimicking IMT from the onset because of the relatively rare presentation of multiple lesions and lymphadenopathy.

**TABLE 2:** Reported cases of malignant lymphoma mimicking inflammatory myofibroblastic tumor.

Case	Age (years)/sex	Type of lymphoma	Lesion site	Diagnosis on first biopsy	Diagnosis on second biopsy	References	Year of reporting
1	42/F	B cell lineage (unknown details)	Brain	IMT	B cell lineage	15	1996
2	78/F	Marginal zone B‐cell lymphoma	Buccal mucosa	Marginal zone B‐cell lymphoma	‐	16	2008
3	37/M	Hodgkin lymphoma	Mediastinum	IMT	Hodgkin lymphoma	14	2019
Present case	20/M	Hodgkin lymphoma	Mediastinum	IMT	Hodgkin lymphoma		

Abbreviations: IMT, inflammatory myofibroblastic tumor; MZBL, marginal zone B cell lymphoma.

Among the histological CHL subtypes, nodular sclerosis CHL predominates in primary mediastinal lesions. Most patients experience constitutional symptoms (B symptoms) including fevers, night sweats, and weight loss.^17^ Our patient exhibited a mediastinal mass accompanied by multiple lymph node enlargement along with B symptoms. Pathological findings of CHL encompass atypical cells positive for CD15 and CD30, referred to as HRS cells, which are diagnostic hallmarks of CHL. Anaplastic large cell lymphoma cells resemble HRS cells and are positive for ALK and CD30.^18^ In our patient, the diagnosis of CHL was based on tumor cells being positive for CD15, but negative for ALK.

In conclusion, our patient was initially diagnosed with IMT, which was rediagnosed as CHL. In patients with IMT with unfavorable treatment response, it is important to consider possible ML and perform additive biopsy for proper diagnosis.

## AUTHOR CONTRIBUTIONS

KA wrote the manuscript draft. K. Yamasaki, YC, TK, HD, TM, YH, CN, SS, and K. Yatera critically revised the manuscript draft. All authors read and approved the final manuscript.

## CONFLICT OF INTEREST STATEMENT

The author report no conflict of interest.
